# The value of Fast Dixon combined with deep learning technology in contrast agent-free high-resolution magnetic resonance imaging of the brachial plexus

**DOI:** 10.3389/fneur.2025.1558927

**Published:** 2025-06-04

**Authors:** Si Xie, Xuehui Du, Zhitao Zhang, Guangxiang Chen

**Affiliations:** ^1^Department of Radiology, The Affiliated Hospital of Southwest Medical University, Luzhou, China; ^2^Siemens Healthineers, Shanghai, China

**Keywords:** brachial plexus, magnetic resonance imaging, Fast Dixon, deep learning, contrast agent-free

## Abstract

**Introduction:**

This study aimed to investigate the application of Fast Dixon combined with the deep resolve gain (DRG) technique for enhancing the image quality of the brachial plexus on high-resolution MRI without the use of contrast agents.

**Methods:**

Heavily T2–weighted Fast Dixon high-resolution coronal thin-slice magnetic resonance imaging was conducted on 19 social volunteers. Post-scan, the original data underwent reconstruction using deep learning-based denoising technology. Subjective quality scores were assigned to both the original and MIP images, and those processed with and without denoising technology were compared. The signal-to-noise ratio (SNR) and contrast-to-noise ratio (CNR) values for each segment of the bilateral brachial plexus were measured and analyzed to assess image quality.

**Results:**

Subjective evaluations revealed that the quality of both original thin-slice and thin-MIP images processed with the DRG significantly outperformed those processed without the DRG (original thin-slice *p* = 0.005, thin-MIP *p* < 0.05). The bilateral SNRs and CNRs of each anatomical structure (root, trunk, cord, branch) of the brachial plexus in the experimental group with DRG were significantly greater than those in the control group without DRG (*p* < 0.05), as follows: the SNRs of the bilateral nerve roots increased by 35.1–36.2%, the SNRs of the bilateral nerve trunks increased by 40.6–40.8%, and the SNRs of the bilateral nerve cords and branches increased by about 40–45%. The CNR of the bilateral nerve roots increased by 43.1–44.6%, the CNR of the bilateral nerve trunks increased by 41.8–41.7%, and the CNR of the bilateral nerve cords and branches increased by 47.3–50.6% (root *p* < 0.001, trunk *p* < 0.001, cord *p* = 0.001, branch *p* = 0.011).

**Conclusion:**

Fast Dixon T2WI can enhance the visibility of brachial plexus segments to a certain extent through DRG denoising technology, which may be an effective means to visualize the brachial plexus without contrast agent.

## 1 Introduction

The brachial plexus consists of the C5–C8 cervical and T1 thoracic nerve roots, traversing between the anterior and middle scalene muscles. Small branches of the plexus are located behind the pectoralis minor muscle and are distributed in the axilla, scapular region, and medial bicipital groove (1, 2). Its deep anatomical location, complex trajectory, and proximity to adjacent blood vessels present significant challenges in acquiring high-quality magnetic resonance imaging (MRI) of this structure (3).

Currently, two principal approaches are employed for brachial plexus MRI. The first is nerve imaging, which is based on diffusion-weighted imaging (DWI), leveraging the restricted movement of water molecules caused by the nerve myelin sheath and perineurium, which generates high signals on DWI (4). Additionally, diffusion tensor imaging (DTI), derived from DWI, enables visualization of the brachial plexus as fiber bundles (5, 6). The second approach relies on T2–weighted imaging, which uses differences in transverse relaxation between low-protein water molecules in the endoneurium and surrounding tissues. This method accentuates the signal from nerve fiber bundles, delineating the structural morphology of peripheral nerves (7). Among the available techniques, 3D TSE-STIR remains the clinical mainstay for brachial plexus imaging. While this sequence demonstrates substantial potential in nerve trajectory visualization through multiplanar reconstruction and maximum intensity projection (MIP) (8, 9), it faces critical limitations in radiofrequency field (RFF) homogeneity, particularly at higher field strengths. These B1 inhomogeneities frequently induce localized fat suppression failure (10), where residual fat signals obscure critical neural structures, compromising diagnostic confidence in anatomically complex regions. To address this technical limitation, the present study introduces the Fast Dixon T2WI sequence, which combines water-fat separation algorithms for enhanced background suppression, thereby improving nerve-to-background contrast, with thin-slice, zero-gap acquisition that enables high-resolution, high-contrast quasi-three-dimensional imaging of the brachial plexus without the use of exogenous contrast agents. Although the reduction in voxel volume leads to a decrease in the original image’s signal-to-noise ratio (SNR), this drawback is mitigated through the integration of an artificial intelligence (AI)-based deep learning denoising technique, thereby enhancing the overall image quality of the brachial plexus.

For MR images, the main noise sources are the electronic noise generated in the signal receiving circuit and the dielectric and inductive coupling effects caused by the conducting medium in the body. The SNR refers to the ratio of the MR signal-to-noise, which is the core parameter used to measure the quality of MR images. The traditional methods used to improve the SNR include changing the repetition time (TR), echo time (TE), FOV, basic resolution, flip angle (FA), bandwidth, etc., but are usually accompanied by a certain extent of scan time extension. The traditional approaches for improving the signal-to-noise ratio (SNR) involve parameter adjustments such as repetition time (TR), echo time (TE), field of view (FOV), matrix size, flip angle (FA), and bandwidth, but these modifications typically incur prolonged scan durations. With the rapid development of deep learning algorithms in the field of medical imaging, the reconstruction of physical–mathematical constraints on MR images has overcome the triangular challenges of the resolution, SNR, and acquisition time speed in traditional reconstruction. Its application has now appeared in various research publications (11–15). The deep resolve gain (DRG) algorithm in this study leverages its inherent capability for noise reduction without requiring additional scanning time (15).

## 2 Materials and methods

### 2.1 General information

From July 2023 to December 2024, 19 healthy social volunteers, including 15 males and 4 females, participated in the study. The age range of the participants was 23–58 years, with a mean age ± standard deviation of 33.79 ± 7.72 years.

Inclusion Criteria: Eligible participants were healthy volunteers with no documented history of brachial plexus pathology, including traumatic injury, inflammatory conditions, neoplastic involvement, or congenital anomalies. Candidates were required to demonstrate normal sensorimotor function of the upper extremities and to be free from systemic comorbidities that could potentially impact neurological integrity, such as diabetes mellitus, autoimmune diseases, or metabolic disorders. All participants met MRI safety requirements, specifically the absence of pacemakers, metallic implants, or claustrophobia, and provided written informed consent.

Exclusion criteria: Exclusion criteria encompassed any history of brachial plexus injury, surgical intervention, or radiation therapy; the presence of neurological disorders affecting central or peripheral pathways, including carpal tunnel syndrome or demyelinating diseases; administration of neurotoxic medications within the preceding 6 months; chronic alcohol dependence; biochemical deficiencies compromising neural health; anatomical or physiological factors likely to compromise image quality, such as artifact-generating non-ferromagnetic implants; current pregnancy or lactation; and enrollment in other clinical trials within the last 30 days.

### 2.2 Equipment and methods

All the scanning protocols were conducted using a Siemens Magnetom Vida 3.0T magnetic resonance scanner, software version XA50, equipped with a 20–channel head and neck combined coil and an Ultra Flex18 small flexible coil. Prior to the examination, the participants were briefed on potential noise and heat sensations associated with the procedure to facilitate their cooperation.

During the scans, the participants were placed in a supine position with their head entering the scanner first. An Ultra Flex18 small flexible coil was placed to cover the junction of the shoulder and neck, as well as the axillary region. Magnetic resonance sandbags were symmetrically positioned on the dorsal side of the upper arms to enhance the display of distal brachial plexus signals.

A direct coronal non-interval scan was performed, covering the region from the C3 vertebral level to the humeral head in the superior–inferior direction and from the anterior edge of the vertebral body to the posterior edge of the spinal canal in the anterior–posterior direction.

The scanning parameters for the T2WI Fast Dixon sequence were as follows: repetition time (TR)/echo time (TE) = 6,220 ms/104 ms; turbo factor (ETL) = 21; parallel imaging acceleration factor (GRAPPA) = 2; average = 2; field of view (FOV) = 288 × 360 mm^2^; acquisition matrix = 288 × 448; slice thickness = 1.6 mm (reconstructed in-plane resolution: 0.8 × 0.8 mm^2^); contiguous slices (gap = 0 mm) = 46; and total scan duration = 4 min 46 s.

Upon completing the scans, the deep resolve feature was activated in the advanced reconstruction section of the reconstruction parameter card via the original dataset. The DRG denoising mode was activated, with the denoising intensity factor set to 8 and the enhancement level factor set to 5. The first group of images without DRG technology was used as the control group, while the denoised DRG images were used as the experimental group.

### 2.3 Data measurement and image post-processing

All the data were exported in DICOM format to RadiAnt DICOM Viewer 2023 software for measurement. Regions of interest (ROIs) of appropriate size were placed on the roots, trunks, cords, and branches of the brachial plexus, as well as on the scalene muscles, on both sides of the same thin layer along the course of the brachial plexus. The background signal intensity (SI) was determined as the average value of the four-corner areas of the image. The SIs of the roots, trunks, cords, branches, muscles, and background noise were recorded. On the basis of these measurements, the SNR and CNR for each segment of the left and right brachial plexus were calculated via the following formulas: SNR = nerve SI/background noise SI; CNR = (nerve SI – muscle SI)/background noise SI. Additionally, 19 paired pre- and post-denoising MIP image groups were reconstructed, and all thin-slice datasets were processed with 10 mm maximum intensity projection (MIP).

### 2.4 Image evaluations

Two musculoskeletal radiologists with 3 and 10 years of experience independently evaluated the reconstructed MIP images together with the source images in a random order and were blinded to the imaging parameters and clinical information. Like the previously published scoring system (16), the original thin-layer images were evaluated via a three-point scale: 3 points, no background noise, clear boundaries between nerves and tissues, uniform signals, and no artifact interference. Two points, mild noise, local fluctuations in the nerve signal, identifiable but local fuzzy boundaries between nerve and tissue, and mild artifacts; Score 1, the noise was significant, the structure was blurred, the nerve and tissue boundaries could not be distinguished, and artifacts covered key anatomical regions.

The thin-MIP images of the two groups were evaluated via a 4-point scale to evaluate the root, trunk, bundle, and branch (17): 4 points, the corresponding segments of the brachial plexus were continuous and complete, with clear boundaries with surrounding tissues and no artifacts or noise interference. Score 3: Each segment of the brachial plexus could be observed, and only one root/trunk/cord/branch (such as the T1 root or 1 trunk/bundle/pectoral minor branch) in the corresponding segment was slightly blurred, with mild artifacts and noise interference; Score 2: severe loss of the corresponding segments of the brachial plexus (such as T1 root loss/incomplete display of the inferior trunk/blurred medial fasciculus/visible main branches but blurred bifurcations), unclear tissue boundaries, and moderate artifact noise interference; Score 1: the corresponding segment of the brachial plexus could not distinguish the structure, and the image was severely disturbed by artifact noise. When there was disagreement in blind reviews, discrepancy in assessment was resolved through negotiation, and the final outcome was based on their consensus agreement.

### 2.5 Statistical methods

Data analysis was conducted via SPSSPRO statistical software. The measurement data are presented as the means ±standard deviations (X̅ ± S). The Wilcoxon signed-rank test was employed to compare the quality grading between the original thin-slice brachial plexus images and the corresponding thin-MIP reconstructed nerve segment displays. For the SNR and CNR comparisons across brachial plexus segments, the paired Student’s *t* test was applied when normality assumptions were satisfied (assessed via the Shapiro–Wilk test); otherwise, the Wilcoxon signed-rank test was utilized. A two-tailed *p* value <0.05 indicated statistical significance. Interobserver agreement was evaluated via Cohen’s kappa coefficient, which was interpreted as follows: ≤0.20 (poor), 0.21–0.40 (fair), 0.41–0.60 (moderate), 0.61–0.80 (good), and 0.81–1.00 (excellent).

## 3 Results

### 3.1 Qualitative analysis

**Figure 1:**
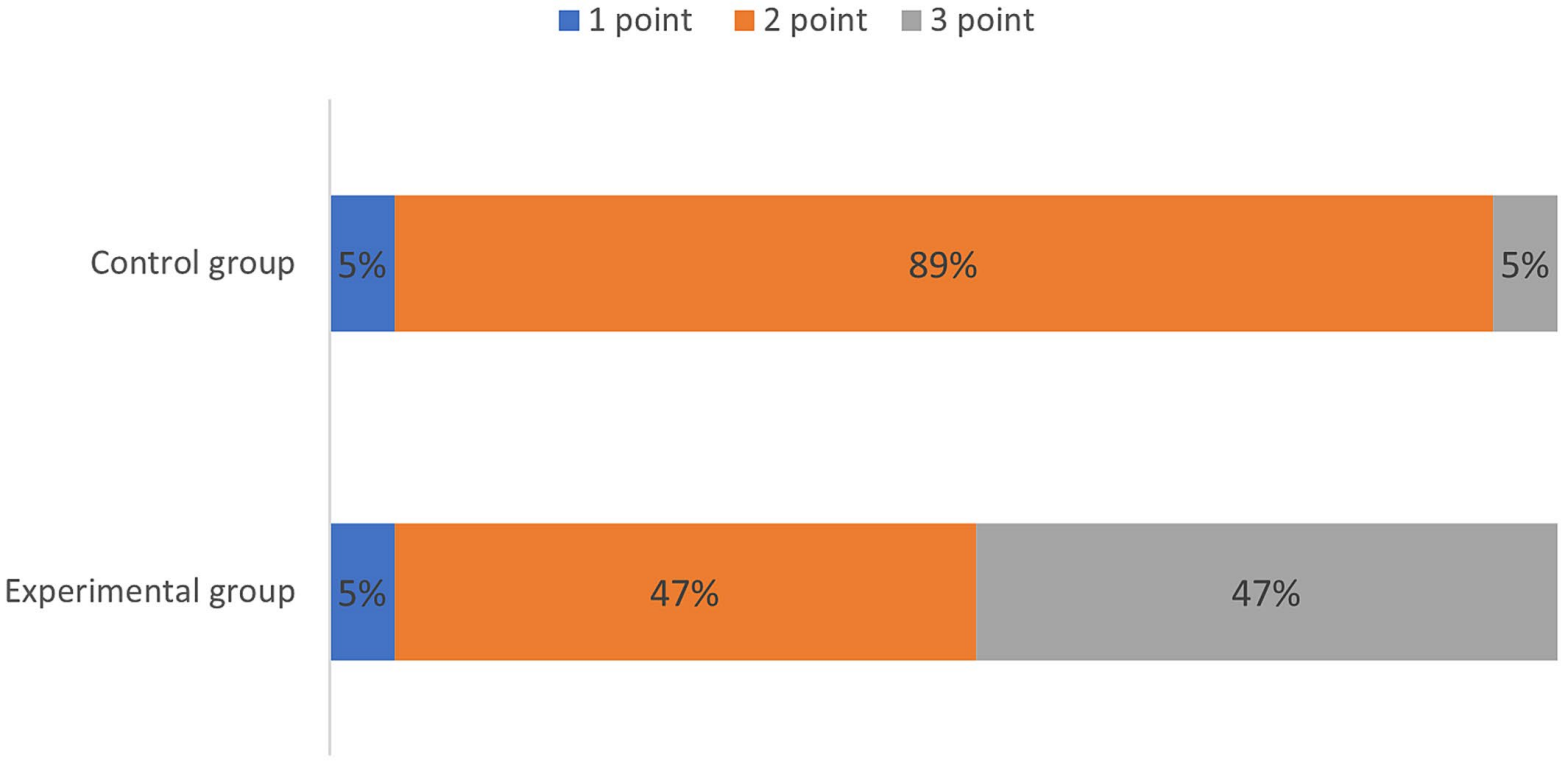
Subjective rating of the original image.

**Figure 2:**
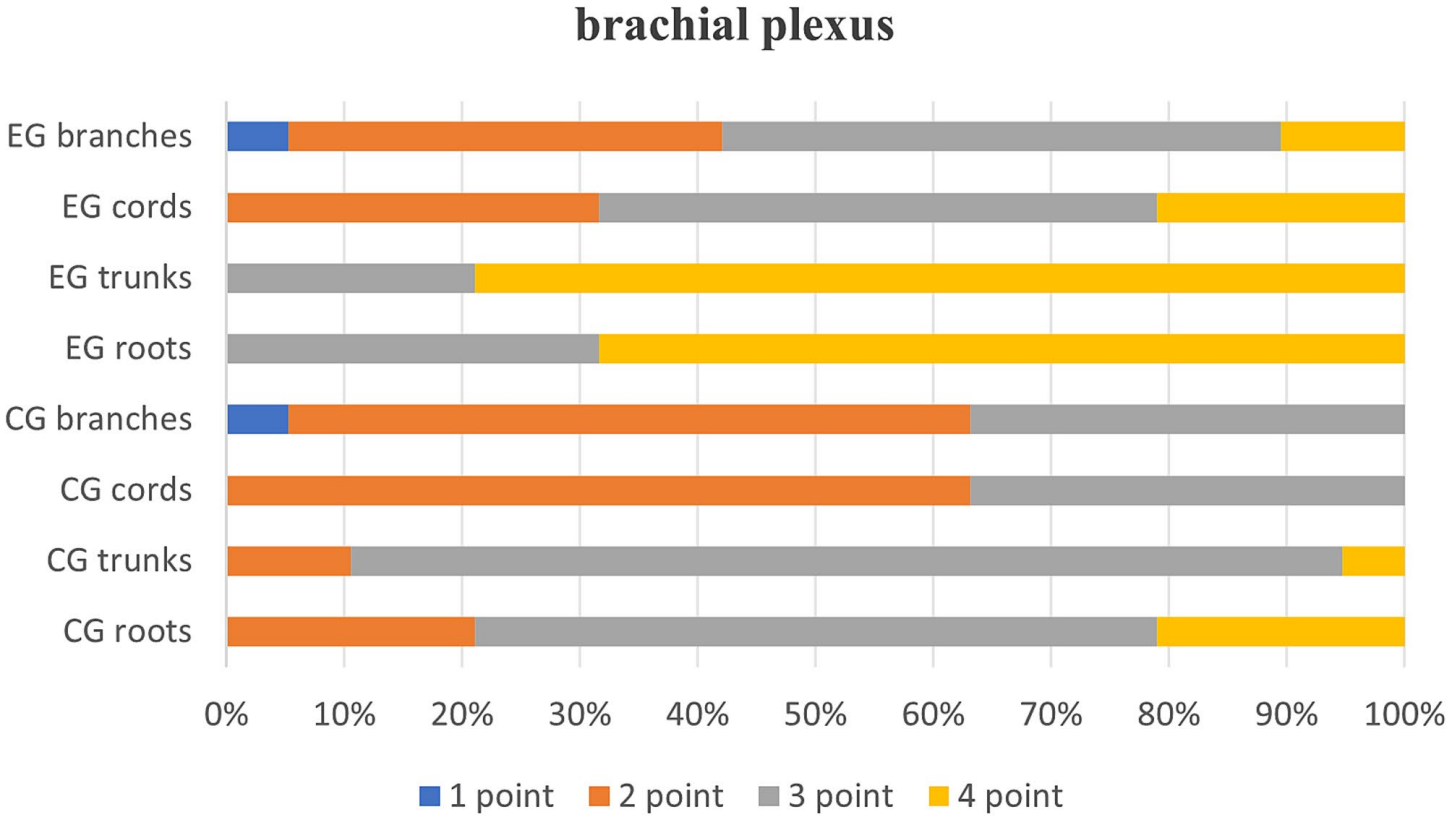
MIP score for each segment of the brachial plexus.

Quantitative scores comparing image quality between two thin-slice images are systematically summarized in Table 1 and are visually represented in Figure 1. Subsequent qualitative evaluations of critical brachial plexus substructures, including roots, trunks, cords, and branch segments, are statistically documented in Table 2, with comparative analyses graphically depicted in Figure 2.

**Table 1:** Comparison of subjective scores for the original images of the brachial plexus between the experimental and control groups.

Score	Control group (*n* = 19)	Experimental group (*n* = 19)	*p*-value
1 point	1	1	0.005**
2 point	17	9	
3 point	1	9	

**p < 0.01.

**Table 2:** Quality evaluation of root, trunk, cord, and branch segments in two groups of post thin-MIP images [*n* = 19, e.g., (%)].

Variables	1 point	2 point	3 point	4 point	*p*-value
CG roots	0 (0)	4 (21)	11 (58)	4 (21)	<0.001**
EG roots	0 (0)	0 (0)	6 (32)	13 (68)	
CG trunks	0 (0)	2 (11)	16 (84)	1 (5)	<0.001**
EG trunks	0 (0)	0 (0)	4 (21)	15 (79)	
CG cords	0 (0)	12 (63)	7 (37)	0 (0)	0.001**
EG cords	0 (0)	6 (32)	9 (47)	4 (21)	
CG branches	1 (5)	11 (58)	7 (37)	(0)	0.011*
EG branches	1 (5)	7 (37)	9 (47)	2 (11)	

*p < 0.05, **p < 0.01. CG, control group; EG, experimental group.

Table 1 qualitatively shows the quality of the thin-layer images of the experimental and control groups. The score of the experimental group was greater than that of the control group, indicating that the quality of the T2WI Fast Dixon image combined with the DRG was better than that of the conventional T2WI Fast Dixon image (*p* = 0.005), and the difference was statistically significant.

Table 2, Quality evaluation of the root, trunk, cord and branch segments of the two groups of images after thin-MIP, *p* < 0.05, indicating that T2WI Fast Dixon images combined with the DRG showed statistically significant differences in the root, trunk, cord and branch of the brachial plexus after reconstruction.

Interobserver agreement was excellent, with Cohen’s kappa values of *k* = 1.000 for the control group and *k* = 0.853 for the experimental group. Thin maximum intensity projection (thin-MIP) images showed good to excellent consistency between the two observers across anatomical segments. In the control group, kappa values for the root, trunk, cord, and branch were *k* = 0.716, 0.835, 0.774, and 0.898, respectively. In the experimental group, the corresponding values were *k* = 0.776, 0.855, 0.883, and 0.897. All differences were statistically significant with *p* < 0.001.

**Figure 3:**
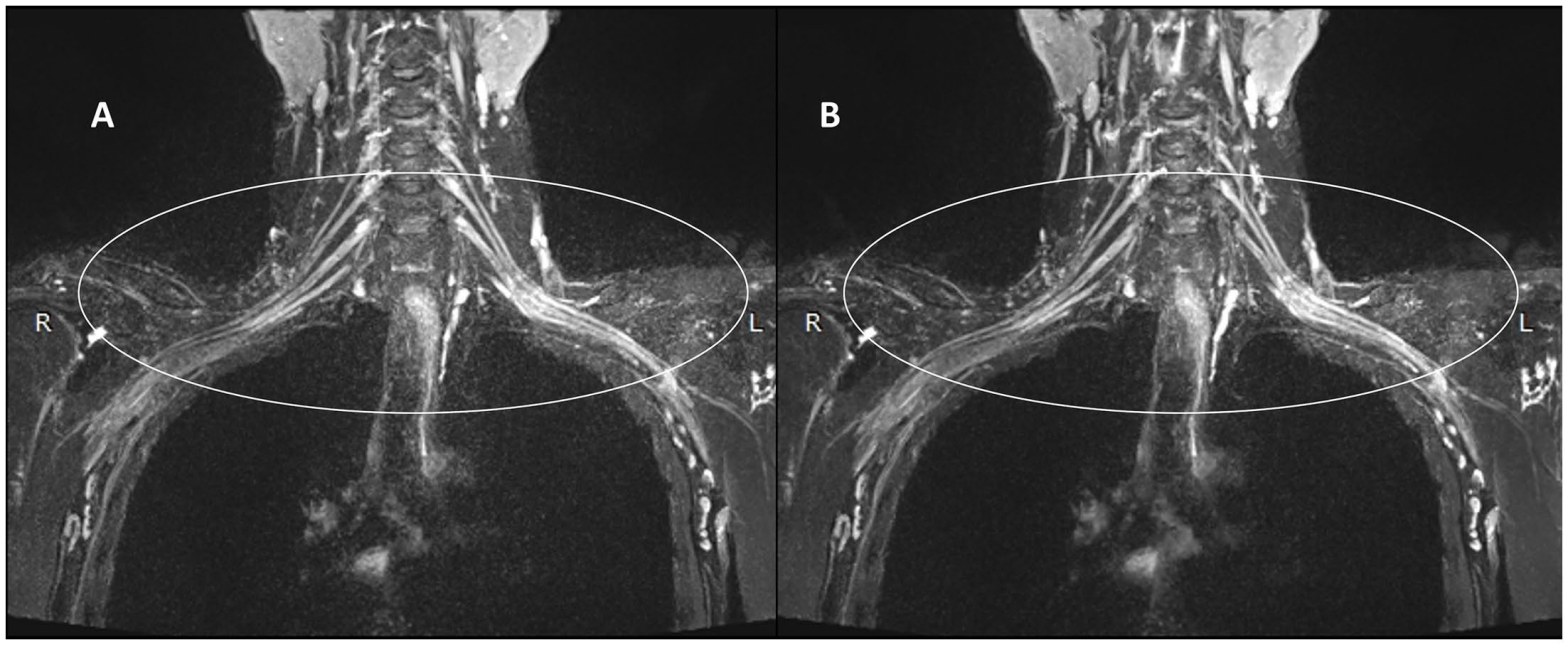
Comparison of the control and experimental groups after the addition of thin-MIP from a 35-year-old male. **A,B** present comparative 10 mm thin-MIP images under identical window width/level settings. **B** (post-DRG) highlight the localized adaptive denoising capability of the DRG-integrated method within central regions (delineated by white circles), which contrasts with **A** (without-DRG) a conventional global filter. The DRG technique significantly improves the SNR and nerve-to-background contrast in anatomically complex areas, such as the cervicothoracic junction and supraclavicular fossa, while maintaining structural fidelity.

**Figure 4:**
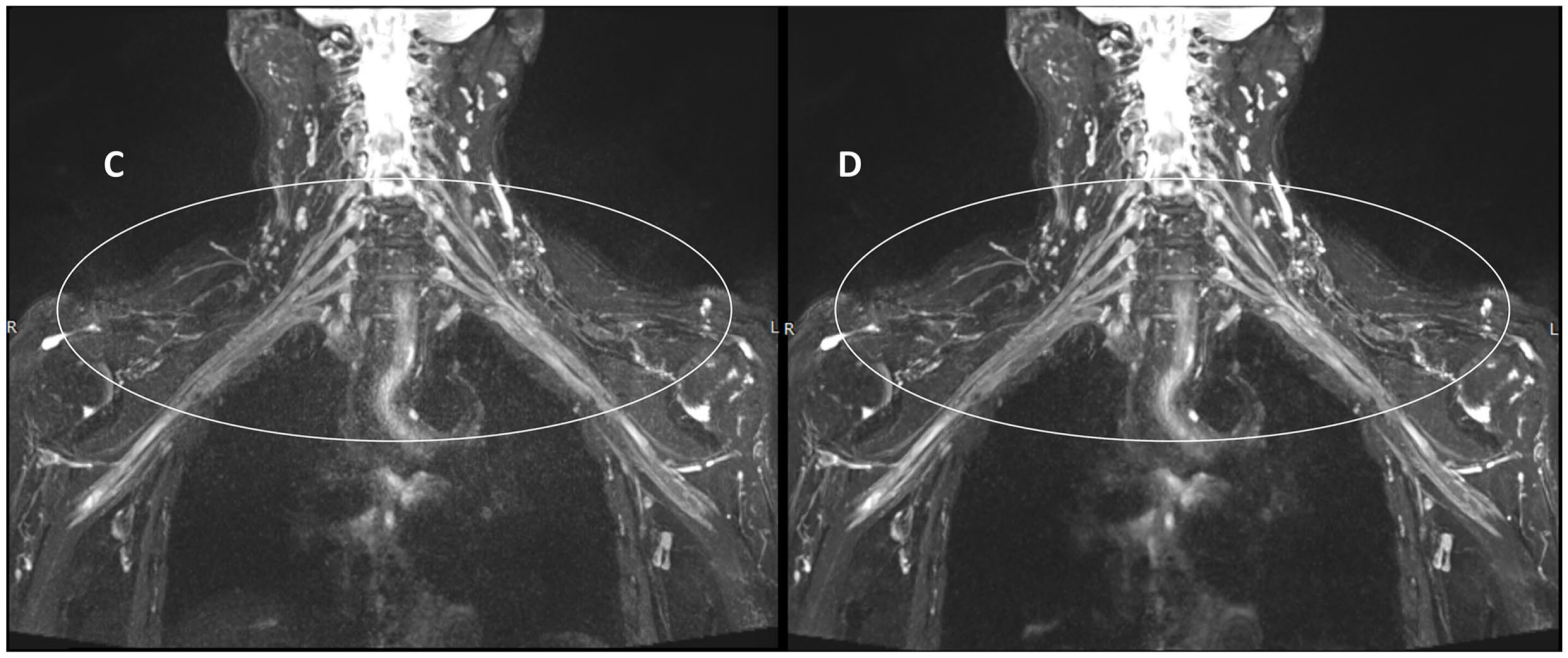
Comparison of the control and experimental groups after thin-MIP from a 59-year-old female. **C,D** present comparative 10 mm thin-MIP images under identical window width/level settings. Compared with **C** (without-DRG), **D** (post-DRG) has better denoising ability and neural background contrast.

Figures 3, 4 present comparative 10 mm thin-MIP images under identical window width/level settings. Panels A/C (without-DRG) and B/D (after-DRG) highlight the localized adaptive denoising capability of the DRG-integrated method within central regions (delineated by white circles), which contrasts with conventional global filtering (A/C). The DRG technique significantly improves the SNR and nerve-to-background contrast in anatomically complex areas, such as the cervicothoracic junction and supraclavicular fossa, while maintaining structural fidelity.

### 3.2 Quantitative analysis

**Figure 5:**
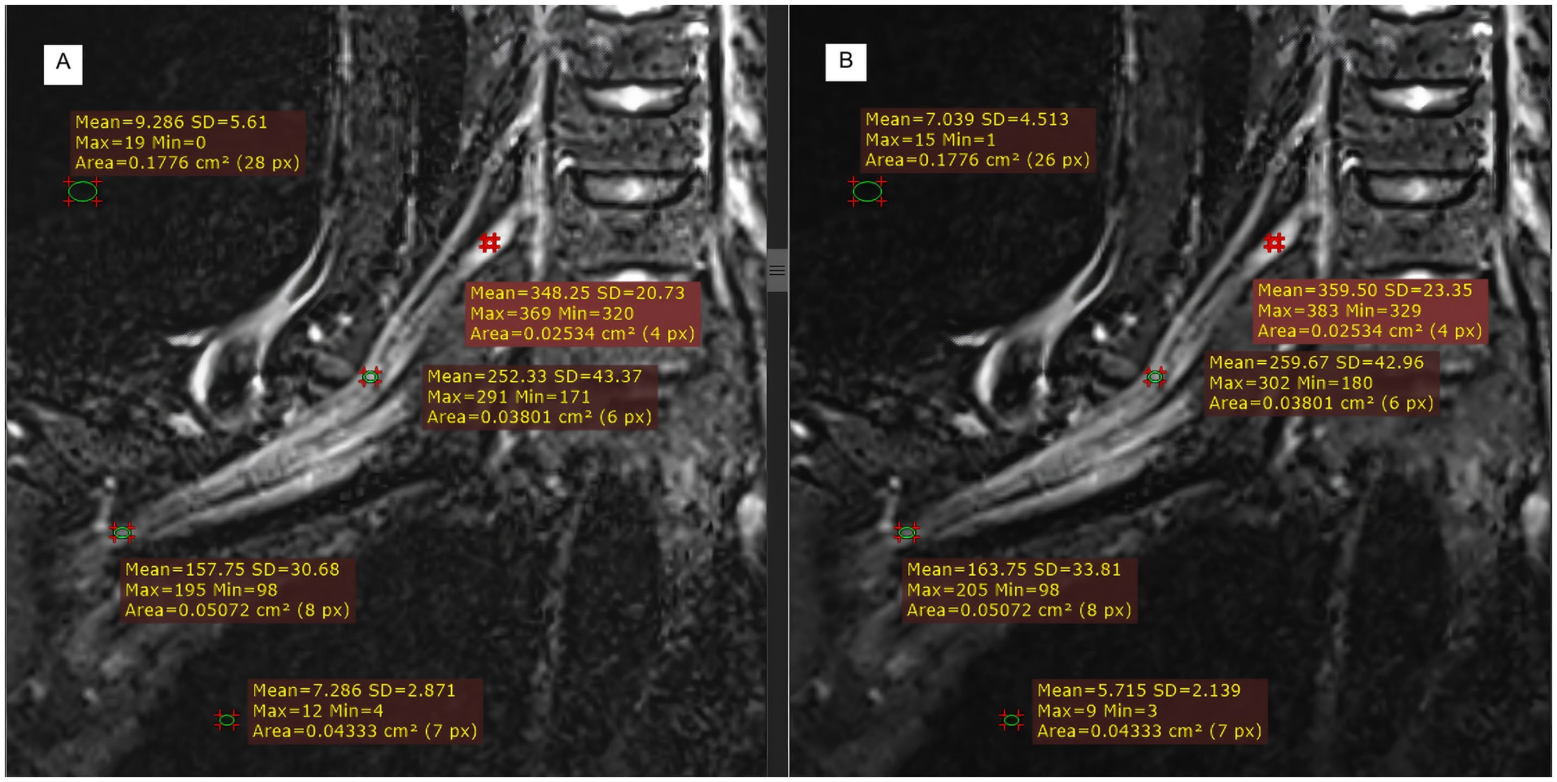
Comparison of T2WI Fast Dixon measurements before and after DRG combination in displaying partial segments of the brachial plexus. **A**: without DRG; **B**: with DRG.

Figure 5 illustrates the measurement scheme for the signal intensities of the right partial segments of the brachial plexus and the background noise in the two groups from another test. Compared with those in the control group (Figure 5A), the signal intensities of the roots, trunks, and cords of the brachial plexus in the experimental group (Figure 5B) were greater, whereas the background noise was significantly lower in the experimental group than in the control group.

**Figure 6:**
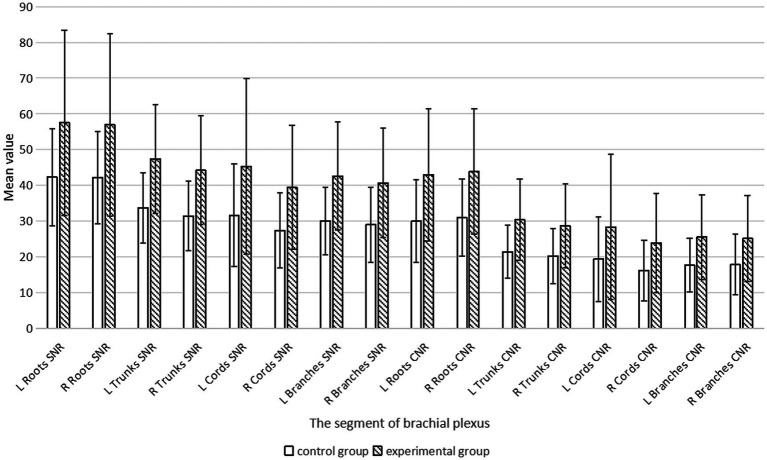
Comparative histogram analysis of the control and experimental groups.

We statistically analyzed the average SNR and CNR values of each segment of the brachial plexus before and after the use of the DRG, and the results are shown in Table 3 and Figure 6. The bilateral SNRs and CNRs of each anatomical structure (root, trunk, cord, branch) of the brachial plexus in the experimental group with DRG were significantly greater than those in the control group without DRG (*p* < 0.05), as follows: the SNRs of the bilateral nerve roots increased by 35.1–36.2% (e.g., left: 57.506 vs. 42.25; right: 56.917 vs. 42.116), the SNRs of the bilateral nerve trunks increased by 40.6–40.8%, and the SNRs of the bilateral nerve cords and branches increased by about 40–45%. The CNR of the bilateral nerve roots increased by 43.1–44.6%, the CNR of the bilateral nerve trunks increased by 41.8–41.7%, and the CNR of the bilateral nerve cords and branches increased by 47.3–50.6% (root *p* < 0.001, trunk p < 0.001, cord *p* = 0.001, branch *p* = 0.011).

**Table 3:** Comparison of SNR and CNR values for each segment of the brachial plexus between the control and experimental groups (x̄ ± s).

Paired variables	x̄±*s*		*p*-value	T value
	Control group	Experimental group		
L roots SNR	42.25 ± 13.526	57.506 ± 25.9	0.002**	No T value
R roots SNR	42.116 ± 12.973	56.917 ± 25.516	0.004**	No T value
L trunks SNR	33.676 ± 9.846	47.353 ± 15.262	<0.001**	−7.868
R trunks SNR	31.381 ± 9.75	44.17 ± 15.233	<0.001**	−7.978
L cords SNR	31.622 ± 14.319	45.302 ± 24.607	<0.001**	No T value
R cords SNR	27.36 ± 10.509	39.422 ± 17.343	<0.001**	No T value
L branches SNR	29.958 ± 9.396	42.54 ± 15.11	<0.001**	No T value
R branches SNR	28.982 ± 10.461	40.657 ± 15.322	<0.001**	−7.307
L roots CNR	29.972 ± 11.515	42.888 ± 18.457	<0.001**	No T value
R roots CNR	30.909 ± 10.803	43.914 ± 17.544	<0.001**	No T value
L trunks CNR	21.399 ± 7.459	30.355 ± 11.364	<0.001**	−7.132
R trunks CNR	20.174 ± 7.706	28.596 ± 11.771	<0.001**	−7.063
L cords CNR	19.344 ± 11.862	28.303 ± 20.325	<0.001**	No T value
R cords CNR	16.153 ± 8.519	23.848 ± 13.948	<0.001**	No T value
L branches CNR	17.68 ± 7.543	25.541 ± 11.87	<0.001**	No T value
R branches CNR	17.775 ± 8.496	25.083 ± 12.073	<0.001**	−5.981

**p < 0.01.

## 4 Discussion

The current approaches to magnetic resonance neuroimaging (MRN) involve the use of high-resolution isotropic 3D imaging to capture the complex pathways of peripheral nerve structures, and sequences with strong T2 weighting or those associated with DWI are used to enhance the signal from nerve tissues while minimizing the signals from surrounding structures. Furthermore, suitable fat suppression techniques are employed to reduce the impact of fat signals (18–20):

1. A brachial plexus nerve display technique based on DWI. This method primarily exploits the principle that water molecules exhibit restricted diffusion, resulting in high signals after the application of a diffusion gradient field, enabling visualization of the brachial plexus nerve. However, it has limitations in terms of spatial resolution and a low signal-to-noise ratio. The diagnostic utility of these images alone is limited, and their combination with other TSE sequences is needed to improve diagnostic efficacy (4).
2. 3D TSE STIR combined with fast spin-echo heavy T2-weighted imaging is a high-resolution technique that enables clear visualization of the brachial plexus nerves and is well-suited for both pre- and post-contrast imaging. In contrast, the T1 and T2 relaxation times of lymph nodes and blood are reduced, potentially affecting their signal characteristics. The longitudinal magnetization vectors of these background signals are suppressed after inversion, resulting in a strong contrast between the brachial plexus and surrounding tissues, which enables clear imaging of the nerves (21, 22). This method is widely used in clinical practice. However, since it involves the administration of a contrast agent, it may not be suitable for patients with poor renal function or those who are allergic to the agent. Additionally, in the neck and shoulder regions, where there is significant anatomical deformation, higher field strengths (especially above 3T) can lead to magnetic field inhomogeneities. This often results in failure of local fat suppression, causing fat signals to obscure the brachial plexus nerve, making it difficult to identify (10). The water-fat separation Dixon technique is relatively insensitive to magnetic field and radiofrequency inhomogeneities, effectively suppressing fat signals and thereby minimizing interference with brachial plexus nerve visualization. However, conventional Dixon sequences are associated with prolonged scan times, increasing susceptibility to motion artifacts and potentially compromising the clarity of anatomical detail.

**Figure 7:**
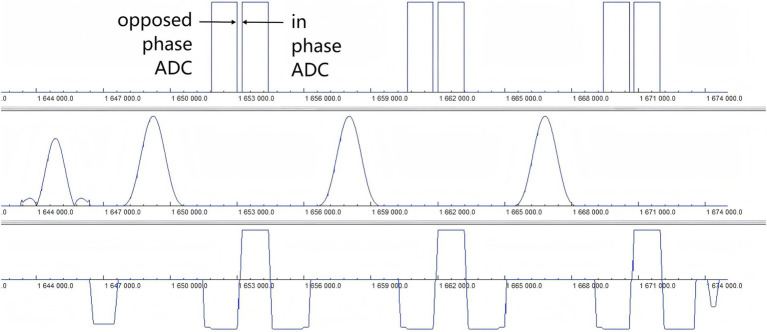
Timing diagram of the T2WI Fast Dixon sequence. The diagram of the Fast Dixon sequence shows opposed phase and in phase echoes acquired in the same repetition time between a pair of refocusing pulses (row 2) and the corresponding readout gradient (row 3). ADC, Analog-to-digital converter.

The Fast Dixon technique modified by the XA version of the manufacturer, which is not sensitive to the inhomogeneity of the main magnetic field and the RFF, was used in this study. The fat signal was completely suppressed, and the display of the brachial plexus was not easily disturbed by the fat signal. Ordinary Dixon (23) completes the acquisition of opposed-phase and in-phase echoes in two anterior and posterior TRs, which are filled in two independent k-spaces, and then water and lipid images are obtained through the automatic calculation of the system to realize the separation of water and fat, and the overall scanning time is relatively long. In this study, Fast Dixon was used to acquire both opposed-phase and in-phase echoes after one TR, as shown in Figure 7. It also merged the damage gradient and readout gradient, increased the amplitude of the gradient, and reduced the ramp time. Therefore, the scanning time of T2WI Fast Dixon is almost half that of conventional T2WI Dixon. In other words, Fast Dixon can achieve higher spatial resolution and average frequency than ordinary Dixon under the same time conditions. Notably, Fast Dixon needs to be activated under high bandwidth conditions. The existence of multiple average times, higher bandwidths and smaller echo intervals also greatly reduces the probability of motion artifacts of the brachial plexus. The same application scenarios also include easy movement tissues such as the neck and pelvic soft tissue. In fact, the T2WI Fast Dixon sequence we used is essentially a 2D sequence, which is less likely to produce motion artifacts than 3D volumetric excitation is. T2WI Fast Dixon can achieve a slice thickness similar to 3D, with better intra-slice resolution than 3D TSE STIR (0.8 × 0.8 m^2^ vs. 1 × 1 m^2^) in the literature (16, 24, 25) and can provide the same interslice resolution, large FOV and post-processing possibilities as 3D STIR TSE. It allows clear visualization of the entire brachial plexus and facilitates structure recognition and image interpretation (26, 27).

This study demonstrates that the T2WI Fast Dixon sequence, integrated with DRG technology, significantly improves brachial plexus visualization through a dual-path architecture guided by deep learning-based noise modeling and signal enhancement, enabling simultaneous noise suppression and enhancement of SNR and CNR. As an autonomously developed intelligent denoising solution by the MR research team, the core innovations of the DRG are as follows: (1) a noise suppression pathway utilizing residual convolutional networks (ResNet) to precisely learn noise distributions (including electronic noise, motion artifacts, and dielectric effects) in low-SNR images (28), achieving joint denoising via synergistic wavelet transform (frequency domain) and pixel-level filtering (spatial domain), coupled with adversarial training (GAN) to generate noise-reduced images highly consistent with authentic high-SNR references (29); (2) a signal enhancement pathway employing attention gates to dynamically amplify local features of critical anatomical structures (e.g., brachial plexus perineurium-epineurium interfaces) while iteratively restoring noise-obscured microstructures. Some prior studies have demonstrated that deep learning via adaptive network architectures can address denoising, resolution enhancement, and scan time reduction (14, 30–32), which is attributable to domain-specific knowledge embedding (e.g., neural pathway patterns) and exploitation of interslice spatial correlations (33, 34), thereby effectively constraining reconstruction challenges in generating high-quality images. Our findings align with the denoising part of the previous study. To address the persistent issue of MRI noise spatial heterogeneity (stemming from SNR depth-dependent attenuation due to receiver coil geometry and regional noise fluctuations induced by parallel imaging techniques) (35, 36), the DRG innovatively incorporates noise maps derived from raw k-space data into the reconstruction pipeline. The noninvasive extraction of noise maps eliminates additional scan time, enabling localized adaptive denoising—targeted suppression in noise-dominant regions (e.g., image centers or motion artifact zones)—while ensuring real-time processing via optimized computational frameworks. Compared with traditional global filters, the DRG markedly improves fat suppression uniformity and nerve-background contrast in anatomically complex regions. Currently, this technology has been extended to multiple domains: optimization of white matter fiber tract visualization in neuroimaging (37), microstructural enhancement of articular cartilage and tendons in musculoskeletal systems (38, 39), and the tissue edge sharpening in body imaging (40). The synergistic “denoising-enhancement” mechanism provides high-confidence imaging, marking a critical step toward the clinical translation of intelligent image reconstruction technologies.

## 5 Limitations

This study has several limitations. First, the sequence used is a non-contrast imaging protocol for the brachial plexus nerve. Some background signals from small blood vessels and lymph nodes are still present, which can slightly affect the display of certain segments of the brachial plexus. In addition, this study employed a single-center design with a relatively small sample size. This limitation may hinder the detection of intergroup differences due to limited sample representativeness, potentially masking true biological or clinical variations and diminishing statistical power. To overcome these constraints, future research should prioritize multicenter collaborations and the inclusion of large-scale cohorts. Multicenter frameworks can integrate geographically diverse populations, thereby enhancing demographic and phenotypic heterogeneity, whereas expanded sample sizes improve statistical power and data robustness, ultimately yielding more comprehensive, generalizable, and statistically conclusive findings. The inclusion of only healthy volunteers also limits its applicability to pathological cases, which may also have contributed to bias in this study. Deep learning performs better than radiomic features in other site case tasks, such as image segmentation, lesion detection, prognosis prediction, and multimodal image registration (41–43), which also makes us full of expectations for the future brachial plexus in processing pathological changes in the brachial plexus. Overall, to improve the robustness of the study results, we should conduct future long-term follow-up studies in the future and need to include more patients with higher acceleration rates and pathological types.

## 6 Conclusion

T2WI Fast Dixon can enhance the visibility of brachial plexus segments to a certain extent through DRG denoising technology, which may be an effective means to display the brachial plexus without contrast agent.

## Data Availability

The original contributions presented in the study are included in the article/supplementary material, further inquiries can be directed to the corresponding author.
